# Evaluating oral swab samples for PRRSV surveillance in weaning-age pigs under field conditions

**DOI:** 10.3389/fvets.2023.1072682

**Published:** 2023-02-03

**Authors:** Onyekachukwu Henry Osemeke, Nathan VanKley, Claire LeFevre, Christina Peterson, Daniel C. L. Linhares

**Affiliations:** ^1^Fieldepi, Iowa State University College of Veterinary Medicine, Ames, IA, United States; ^2^College of Veterinary Medicine, Michigan State University, Lansing, MI, United States; ^3^Carthage Veterinary Service, Carthage, IL, United States

**Keywords:** PRRSV, surveillance, sampling, serum, swabs, weaning-age

## Abstract

**Introduction:**

The use of serum and family oral fluids for porcine reproductive and respiratory syndrome virus (PRRSV) surveillance in weaning-age pigs has been previously characterized. Characterizing more sample types similarly offers veterinarians and producers additional validated sample options for PRRSV surveillance in this subpopulation of pigs. Oral swab sampling is relatively easy and convenient; however, there is sparse information on how it compares to the reference sample type for PRRSV surveillance under field conditions. Therefore, this study's objective was to compare the PRRSV reverse-transcription real-time polymerase chain reaction (RT-rtPCR) test outcomes of oral swabs (OS) and sera samples obtained from weaning-age pig litters.

**Method:**

At an eligible breeding herd, six hundred twenty-three weaning-age piglets from 51 litters were each sampled for serum and OS and tested for PRRSV RNA by RT-rtPCR.

**Results and Discussion:**

PRRSV RT-rtPCR positivity rate was higher in serum samples (24 of 51 litters, 83 of 623 pigs, with a mean cycle threshold (Ct) value of RT-rtPCR-positive samples per litter ranging from 18.9 to 32.0) compared to OS samples (15 of 51 litters, 33 of 623 pigs, with a mean Ct of RT-rtPCR positive samples per litter ranging from 28.2 to 36.9); this highlights the importance of interpreting negative RT-rtPCR results from OS samples with caution. Every litter with a positive PRRSV RT-rtPCR OS had at least one viremic piglet, highlighting the authenticity of positive PRRSV RT-rtPCR tests using OS; in other words, there was no evidence of environmental PRRSV RNA being detected in OS. Cohen's kappa analysis (Ck = 0.638) indicated a substantial agreement between both sample types for identifying the true PRRSV status of weaning-age pigs.

## 1. Introduction

Porcine reproductive and respiratory syndrome virus (PRRSV) is an enveloped single-stranded positive-sense RNA virus belonging to the family Arteriviridae (1, 2). PRRSV is the etiological agent of Porcine Reproductive and Respiratory Syndrome (PRRS), an economically important swine disease costing the United States swine industry hundreds of millions of dollars in annual revenue losses (3). A typical PRRS outbreak in a sow herd is clinically characterized by respiratory difficulties in younger pigs, spikes in reproductive failures in sows, as well as accompanying increases in neonatal losses, pre-weaning mortality, and sow deaths (4–6).

Over the last three decades, the swine industry has significantly evolved in its effort to curb the menace of PRRSV; a variety of producer-driven PRRSV management programs have been instituted (7). A few factors that may have influenced the evolution of the mentioned programs include the growth and increased consolidation of swine enterprises (8), the emergence and reemergence of PRRSV variants (9, 10), improved understanding of the atypical ecology of PRRSV (11–13), improved diagnostic methods (14, 15) and tools (16–18), and the availability and adoption of technologies such as commercial vaccines (19) and air filtration systems (20).

PRRSV surveillance remains an important component of PRRSV management programs (18, 21, 22). Especially at low prevalence, effective PRRSV surveillance in breeding herds can be cost-prohibitive and usually requires a relatively large number of animals to be sampled. This challenge is a major reason aggregate samples (10, 21, 23, 24) have increasingly become the most predominant specimens submitted to US veterinary diagnostic laboratories for PRRSV molecular testing (10).

Weaning-age pigs (typically 2–3 weeks of age) are an epidemiologically important subpopulation for PRRSV control or elimination programs, not only because their PRRSV shedding status reflects the PRRSV status of a breeding herd (25), but they are also often translocated and are vehicles for swine disease pathogen spread.

Serum is the reference specimen to establish the PRRSV active circulation in pig populations (18, 25). With animal welfare concerns, sampling difficulty, needed expertise, and the cost of sampling materials associated with serum sampling, there is a need to evaluate alternative sampling strategies. One alternative is family oral fluids (FOFs) sampling, which has been shown to be a cost-efficient sample type for PRRSV surveillance, especially when PRRSV is at a low prevalence (26). Obtaining a FOF sample, however, requires voluntary interaction between litter mates and a sampling rope (23, 27).

There is therefore a need to evaluate other sampling options for PRRSV monitoring in weaning-age pigs, especially as swine practitioners are already submitting some of these sample types to veterinary diagnostic laboratories for testing of PRRSV and other swine pathogens (10, 28).

Thus, this study sought to compare PRRSV RNA RT-rtPCR detection rates in oral swabs to detection rates in serum samples from weaning-age pig litters.

## 2. Materials and methods

This study was approved by the Iowa State University Institutional Animal Care and Use Committee (IACUC-19-118).

### 2.1. Study design and eligibility criteria

This was a prospective field study conducted in a conveniently identified swine breeding herd that fit the eligibility criteria.

Serum samples and matched oral swabs were collected from all pigs within fifty-one litters. To be eligible, a breeding herd had to be PRRSV-positive unstable based on the American association of swine veterinarians' guidelines (18). Briefly, a PRRSV-positive unstable herd is a herd that tests RT-rtPCR positive for wildtype PRRSV on routine monitoring.

An eligible herd would also have diagnostic evidence of PRRSV circulation and would not have used PRRS virus vaccines during the study or in the previous 2 years.

### 2.2. Sample collection

Oral swabs were collected from each piglet in the study litters by rotating a polyester swab stick (Puritan, ME, USA) along the buccal mucosa between the back of the tongue and inner cheek of manually restrained pigs. Each swab was transferred to separate 2 ml microcentrifuge tubes (Fisher, MA, USA) containing 1 ml of Phosphate Buffered Saline (Gibco, MA, USA).

Serum samples were collected from each piglet in the study litters into 8.5 ml vacutainer tubes (Becton Dickinson, NJ, USA) *via* jugular venipuncture in manually restrained piglets.

All samples were matched by litter, i.e., identified by the sow identification number, stored at 4°C, and transported to an AALVD (American Association of Veterinary Laboratory Diagnosticians)-certified veterinary diagnostic laboratory for PRRSV RNA laboratory investigation by RT-rtPCR tests.

### 2.3. Sample size justification

The sow farm used for this study weaned an average of 238 litters a week. The sample size of 51 litters aimed at having an at least 95% confidence of selecting ≥1 PRRSV-positive litter (a litter having at least one PRRSV-viremic pig) assuming a 5% PRRSV-prevalence, sampling without replacement, and a perfect RT-rtPCR test (29, 30).

### 2.4. Statistical analysis

All statistical calculations and graphing were done on R statistical software (31).

#### 2.4.1. Within litter prevalence and RT-rtPCR mean cycle threshold values

Within litter prevalence (WLP) by OS, and by serum for each litter were separately calculated as the proportion of piglets within a litter that tested RT-rtPCR positive for OS, and serum respectively.


(1) $$\begin{array}{l} WLP=\frac{Number\;of\;RT-rtPCR\;positive\;piglets\;in\;litter}{Total\;number\;of\;piglets\;in\;litter} \end{array}$$

A plot of OS WLP by serum WLP was made using the ggplot2 package (32) on the R statistical software (31).

The mean cycle threshold (Ct) value for RT-rtPCR PRRSV positive samples for both sample types for each sampled litter was calculated as:


(2) $$\begin{array}{l} Mean\;Ct=\frac{\overset{n_{pos}}{\underset{i=1}{\sum }}\left(Ct_{pos}\right)}{n_{pos}} \end{array}$$

Where *Ct*_*pos*_ is the Ct for each RT-rtPCR positive sample *i*, and *n*_*pos*_ is the number of RT-rtPCR positive samples within that litter.

The effect of the serum WLP in the *jth* litter on the RT-rtPCR detection of PRRSV RNA in OS (*P*^*OS*^) was characterized using a generalized linear model on the lme4 package (33) on R statistical software (31).


(3) $$\begin{array}{l} logit\left(P_{j}^{OS}\right)=log_{e}\left(\frac{P_{j}^{OS}}{1-P_{j}^{OS}}\right)=\alpha +\beta {*x}_{j} \end{array}$$

$$\begin{array}{l} Consequently,\\ the\;estimated\;probability\;of\;detection\;\left(p\right)\;is\;calculated\;as: \end{array}$$

(4) $$\begin{array}{l} \frac{e^{\alpha +\beta *x_{j}}}{1+\;e^{\alpha +\beta *x_{j}}} \end{array}$$

Where:

$P_{j}^{OS}$ is the observed result of the Bernoulli trial (RT-rtPCR detection of PRRSV RNA in OS from at least one piglet, 1 or not, 0) in litter *j*,

α* is* the model's intercept,

β is the regression coefficient,

*x*_*j*_ is the WLP in litter *j*, and

*p* is the estimated probability of detection.

#### 2.4.2. Diagnostic performance assessment and Cohen's kappa analysis

Two by two contingency tables (Table 1) were constructed to compare litter-level PRRSV detection by sample type in each litter. When at least one piglet tested PRRSV RT-rtPCR positive by OS or serum, that litter was considered positive for that sample type. A litter was considered truly PRRSV-positive if serum from at least one piglet tested RT-rtPCR positive. Serum RT-rtPCR results, therefore, were considered as the reference in assessing the litter-level sensitivity and specificity of OS.

**Table 1:** 2 x 2 tables comparing PRRSV RT-rtPCR detection in oral swabs and serum in sampled litters.

		**Serum**		

		**Neg**	**Pos**	
Oral swabs	Neg	True negatives (TN)	False negatives (FN)	V = (TN + FN)
	Pos	False positives (FP)	True positives (TP)	W = (FP + TP)
	X = (TN + FP)	Y = (FN + TP)		Z = (TP + TN + FP + FN)

Sensitivity and specificity were thereafter estimated using the formula:


(5) $$\begin{array}{l} Sensitivity\;=\frac{TP}{Y} \end{array}$$

(6) $$\begin{array}{l} Specificity\;=\frac{TN}{X} \end{array}$$

Cohen's Kappa (*C*_*k*_) analysis (34) was thereafter used to assess the litter-level agreement of PRRSV RT-rtPCR detection in OS and serum samples over chance, using the formula:


(7) $$\begin{array}{l} C_{k}=\frac{observed\;agreement-agreement\;by\;chance}{1-agreement\;by\;chance}\\ =\frac{\left(\frac{TP+TN}{Z}\right)\;-\left(\left({\left(\frac{W}{Z}\right)}^{*}\left(\frac{Y}{Z}\right)\right)+\left({\left(\frac{V}{Z}\right)}^{*}\left(\frac{X}{Z}\right)\right)\right)}{1-\;\left(\left({\left(\frac{W}{Z}\right)}^{*}\left(\frac{Y}{Z}\right)\right)+\left({\left(\frac{V}{Z}\right)}^{*}\left(\frac{X}{Z}\right)\right)\right)} \end{array}$$

The scale (23) used to interpret the C_*k*_ is:

0 = *no agreement*

0 < *C*_*k*_ < 0.2 = *slight agreement*

0.21 ≤ *C*_*k*_≥0.40 = *fair agreement*

0.41 ≤ *C*_*k*_≥0.60 = *moderate agreement*

0.61 ≤ *C*_*k*_≥ 0.80 = *substantial agreement*

0.81 ≤ *C*_*k*_≥1.00 = *almost perfect agreement*

## 3. Results

A 5,300-head commercial breed-to-wean farm in the Midwestern United States was selected for this study based on eligibility criteria, and this study was conducted in the third quarter of 2021. At the time of the study, the herd was within 3 months of a PRRSV-2 outbreak with the PRRSV outbreak strain having an open reading frame 5 (ORF5) sequence restriction fragment length polymorphism (RFLP) pattern of 1-7-4.

Weaning-age pigs between 18 and 21 days of age belonging to fifty-one litters were conveniently selected, and both oral swabs and serum samples were collected. There was a total of 623 piglets across the 51 sampled litters.

### 3.1. Within-litter prevalence

Across all study litters, the within-litter prevalence ranged from 0.00 to 57.14% for oral swabs, and 0.00 to 100.00% for serum samples. There were more pigs testing RT-rtPCR positive by serum (83/623) than by OS (33/623).

**Figure 1:**
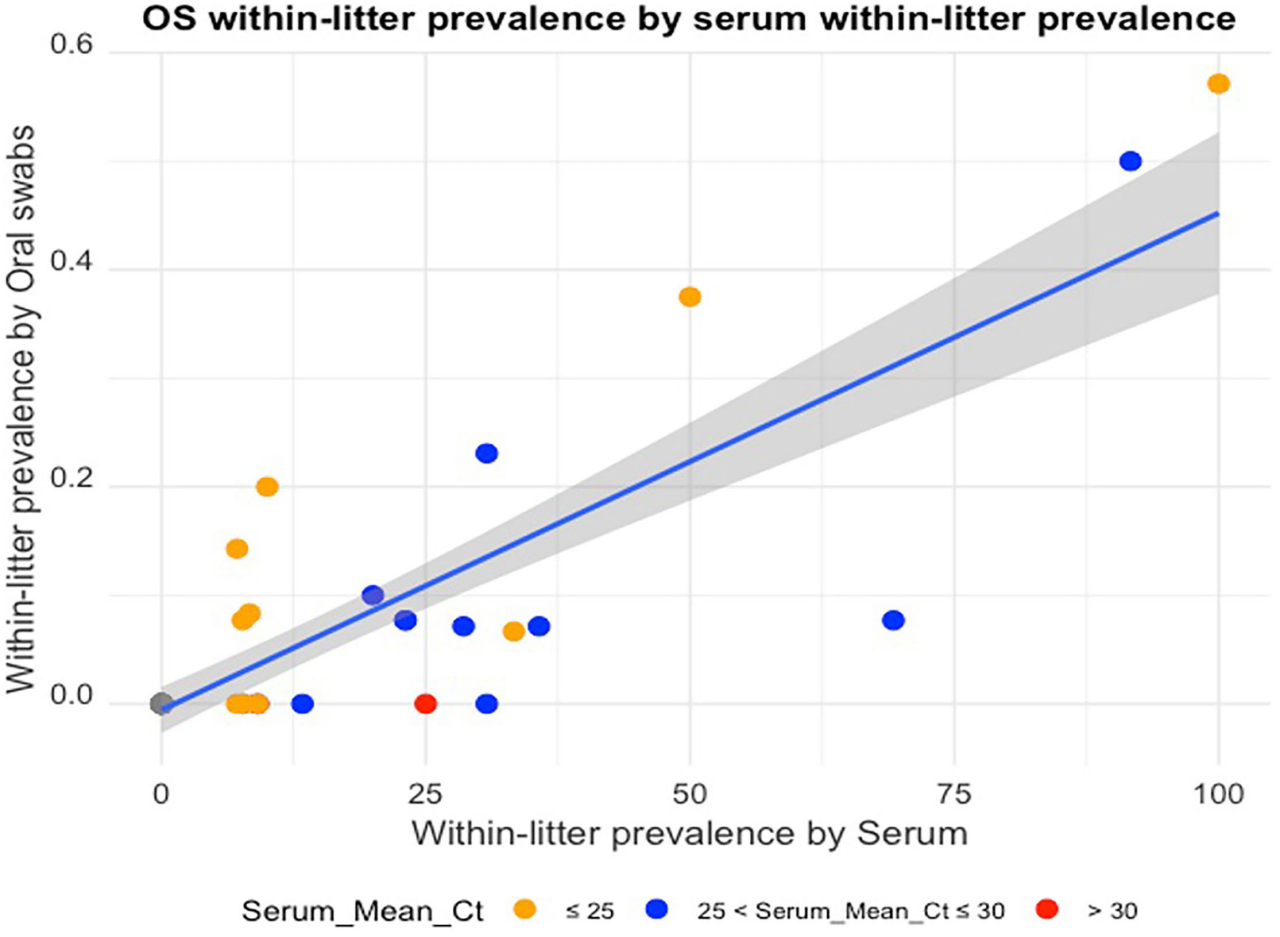
Oral swab within-litter prevalence plotted against serum within-litter prevalence, with colors indicating mean Ct ranges of the RT-rtPCR-positive serum samples for each litter.

The mean Ct of RT-rtPCR positive samples per litter ranged from 28.2 to 36.9 for OS samples, and 18.9 to 32.0 for serum samples. When the mean Ct of the PRRSV-positive serum samples for a litter was over 30 (*n* = *2*), there was no OS from that litter testing PRRV-positive by RT-rtPCR. There was a positive linear relationship between the OS WLP and serum WLP (Figure 1) with a Pearson correlation coefficient value of 84.57%. Table 2 shows the actual WLP values by OS and serum and the mean Ct values of RT-rtPCR PRRSV-positive OS and serum for each litter (identified by sow number).

**Table 2:** Within-litter prevalence (WLP) and mean cycle threshold values for serum and oral swab (OS) sample types for each litter sampled.

**Litter (sow) ID**	**Serum positivity rate**	**OS positivity rate**	**Serum WLP**	**OS WLP**	**Serum mean Ct**	**OS mean Ct**
44816	0/15	0/15	0%	0%	-	-
45574	1/11	0/11	9%	0%	28.20	-
45590	11/12	6/12	92%	50%	26.79	33.95
46160	0/12	0/12	0%	0%	-	-
46419	0/13	0/13	0%	0%	-	-
46585	3/13	1/13	23%	8%	28.70	34.40
46829	9/13	1/13	69%	8%	29.48	36.00
47422	0/12	0/12	0%	0%	-	-
47803	1/13	1/13	8%	8%	22.50	30.30
47922	0/10	0/10	0%	0%	-	-
48173	0/9	0/9	0%	0%	-	-
48920	1/11	0/11	9%	0%	32.00	-
49178	0/14	0/14	0%	0%	-	-
49266	0/14	0/14	0%	0%	-	-
49285	0/9	0/9	0%	0%	-	-
49305	1/14	2/14	7%	14%	24.00	33.50
49328	0/11	0/11	0%	0%	-	-
49415	3/13	1/13	23%	8%	29.80	28.20
49556	0/11	0/11	0%	0%	-	-
50396	2/15	0/15	13%	0%	29.45	-
50408	4/13	0/13	31%	0%	28.30	-
50555	0/14	0/14	0%	0%	-	-
50607	0/14	0/14	0%	0%	-	-
50715	14/14	8/14	100%	57%	21.04	32.29
50793	0/14	0/14	0%	0%	-	-
50797	1/13	0/13	8%	0%	27.40	-
50828	0/12	0/12	0%	0%	-	-
50888	1/14	0/14	7%	0%	18.90	-
51305	2/10	1/10	20%	10%	29.10	36.80
51374	0/14	0/14	0%	0%	-	-
51651	0/13	0/13	0%	0%	-	-
51857	0/13	0/13	0%	0%	-	-
51860	4/14	1/14	29%	7%	26.33	34.60
51866	4/8	3/8	50%	38%	24.28	34.17
51873	5/15	1/15	33%	7%	20.78	36.90
52062	0/11	0/11	0%	0%	-	-
52155	0/14	0/14	0%	0%	-	-
53026	0/14	0/14	0%	0%	-	-
53072	0/7	0/7	0%	0%	-	-
53102	1/10	2/10	10%	20%	18.90	31.70
53249	0/11	0/11	0%	0%	-	-
53294	4/13	3/13	31%	23%	25.25	34.43
53325	0/9	0/9	0%	0%	-	-
53358	1/13	0/13	8%	0%	25.00	-
53364	0/12	0/12	0%	0%	-	-
53372	0/9	0/9	0%	0%	-	-
53386	1/11	0/11	9%	0%	21.60	-
53451	3/12	0/12	25%	0%	32.00	-
53485	5/14	1/14	36%	7%	28.84	35.40
54933	1/12	1/12	8%	8%	23.00	34.40
54941	0/11	0/11	0%	0%	-	-

**Figure 2:**
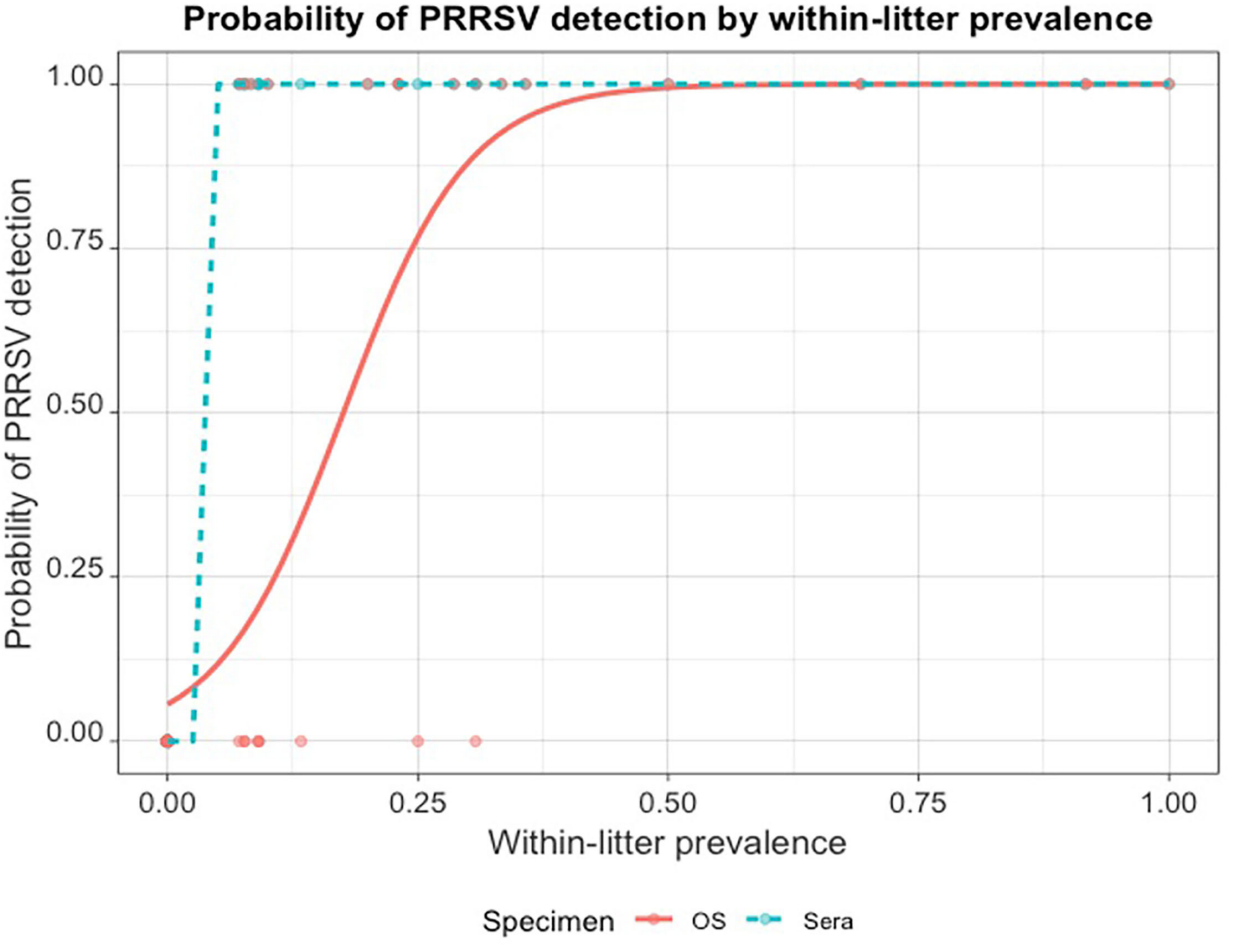
Probability of PRRSV RT-rtPCR detection in at least one pig when litters are fully sampled by oral swabs (OS) compared to within-litter prevalence established by serum testing.

From the regression output, the model's intercept (lower and upper 95% confidence intervals) was −2.82 (−4.420, −1.661) , and the regression coefficient (lower and upper 95% confidence intervals) was 0.16 (0.085, 0.268). Thus, the odds of detecting at least one PRRSV-positive piglet by RT-rtPCR in an OS sampled litter increased by *e*^0.16^ *or* 1.174 (*or* 17.4%) for every unit (1%) increase in WLP; the *p*−*values* for both coefficients were < 0.001. The probability of detecting at least one PRRSV-positive piglet by RT-rtPCR in OS rapidly increased as the within-littler prevalence increased (Figure 2).

### 3.2. Diagnostic performance assessment and Cohen's kappa analysis

A tabular comparison of the litter-level PRRSV RNA Rt-rtPCR detection using both sample types is shown in Table 3. Using equations (5) and (6), sensitivity and specificity of OS for fully sampled litters was 62.5% and 100%, respectively.

**Table 3:** Comparison of PRRSV RT-rtPCR detection in oral swabs and serum samples from litters of weaning age pigs.

		**Serum**		

		**Neg**	**Pos**	
Oral swabs	Neg	27	9	36
	Pos	0	15	15
		27	24	51

Cohen's kappa analysis yielded a value of 0.638 (*p*−*value* < 0.001), indicating a substantial agreement between both sample types for identifying the true PRRSV-status of a fully sampled litter of weaning-age pigs.

## 4. Discussion

Oral swab sampling is a quick and non-invasive sampling technique that has been used in human populations for the detection/diagnosis of tuberculosis (35), Ebola (36), enteric viruses (37), streptococcosis (38), and more recently, Covid-19 (39–41). It has also been used extensively in animal disease surveillance for the detection of Influenza A virus in avian species (42), Bovine Viral Diarrhea virus (43), Foot and Mouth Disease virus (44), Peste des petits ruminants virus (45), Herpesvirus (46), and Nidoviruses (47), amongst others.

Oral swabs have also been used for PRRSV surveillance in swine herds, specifically, their use has been demonstrated in post-weaning age animals (48, 49).

Despite the epidemiological significance of the weaning age pig subpopulation in PRRSV control/elimination programs, serum samples and FOFs are the only two sample types validated specifically for this subpopulation (18, 24, 25). Given that there are potential constraints with the use of these tools, it is no surprise that swine practitioners opt for using other sample types, or use the validated tools conveniently rather than as recommended. Investing in a surveillance program using a sample type that has not been experimentally validated is not justifiable, as one cannot confidently interpret the laboratory results from testing these samples, coupled with the risks associated with a false negative test result.

This study evaluated the suitability of PRRSV RT-rtPCR testing on OS samples as a PRRSV surveillance tool in weaning age pig populations. From the findings of this study, RT-rtPCR tests on serum samples were consistently more likely to give a positive result than RT-rtPCR tests on OS samples for matched litters; this agrees with a previous study (49) that demonstrated that pigs could be viremic and not have detectable quantities of PRRSV in oral swab samples. In addition to this, all positive results from OS samples came from litters containing at least one viremic pig. This gives some credence to this sample type and supports that positive RT-rtPCR tests from OS samples are likely to come from only truly PRRSV-positive litters having at least one viremic pig.

Cycle threshold values from RT-rtPCR tests are a good proxy to estimating the amount of viral RNA present in a sample (50). The observed absence of detectable quantities of PRRSV RNA in oral swabs from a litter when the mean Ct values of RT-rtPCR tests on serum samples from the same litter was >30 (relatively low viremia), suggests that a negative PRRSV RT-rtPCR test on OS may reflect low to no viremia in a sampled pig or litter.

Given that the reasons for evaluating alternative sample types include providing a convenient, animal-welfare friendly, and resource-savvy substitute to serum sampling, a logical next step would be to ascertain an appropriate sample size for OS samples that will provide the same probability of demonstrating disease freedom as serum samples for the same set of statistical assumptions. The design of the current study poses a challenge with determining sample size estimates as this was not the aim of the study; therefore, further studies may be needed in which samples will be matched by individual piglets, sourced from multiple herds, and include more sample-types for pairwise comparisons. Also, litters were conveniently selected and do not necessarily represent the status of other non-sampled litters. Therefore, the true PRRSV-prevalence in the herd could not be calculated with this dataset.

For the current study, when serum samples were first collected from the piglets, there was difficulty in obtaining oral swabs from those piglets as the buccal mucosa had often dried out, likely from vocalization. It is possible this affected the findings of the study, and the authors acknowledge this as a limitation; the design of future studies will be refined to minimize this occurrence.

In real world PRRSV surveillance scenarios for pathogen detection, sampling entire members of multiple litters using either sample type is cost-prohibitive and impractical; the purpose of sampling entire litter members was to facilitate an understanding of how the pressure of PRRSV infection within a litter influenced the probability of detecting at least one viremic pig by RT-rtPCR on OS samples. This was successfully characterized.

## 5. Conclusion

This study evaluated the use of OS under field conditions for PRRSV RNA detection by RT-rtPCR, as compared to serum samples. OS sampling offers a welfare-friendly, resource-savvy, easier, specific, but less sensitive alternative for PRRSV surveillance in weaning-age pig populations, negative RT-rtPCR results using this sample type should be interpreted with caution.

Further studies are needed to characterize the recommended sample size of OS needed to match serum sampling sensitivity for PRRSV surveillance.

## Data availability statement

The original contributions presented in the study are included in the article/supplementary material, further inquiries can be directed to the corresponding author.

## Ethics statement

The animal study was reviewed and approved by Institutional Animal Care and Use Committee. Written informed consent was obtained from the owners for the participation of their animals in this study.

## Author contributions

DL, NV, and CL conceptualized the study. NV and CL carried out the sampling. OO and DL carried out the statistical analysis. NV, OO, CL, and DL discussed the output of all the analyses. CP and OO reassessed the findings of the study. OO drafted the first manuscript. All authors proofread, contributed to- and approved the final version of the manuscript.
